# CO_2_ adsorption in Y zeolite: a structural and dynamic view by a novel principal-component-analysis-assisted *in situ* single-crystal X-ray diffraction experiment

**DOI:** 10.1107/S2053273318017618

**Published:** 2019-02-06

**Authors:** Eleonora Conterosito, Luca Palin, Rocco Caliandro, Wouter van Beek, Dmitry Chernyshov, Marco Milanesio

**Affiliations:** a Dipartimento di Scienze e Innovazione Tecnologica, Università del Piemonte Orientale, Via Michel 11, Alessandria 15121, Italy; b Nova Res s.r.l., Via D. Bello 3, Novara 28100, Italy; c Institute of Crystallography, CNR, via Amendola 122/o, Bari 70126, Italy; d Swiss–Norwegian Beamlines, ESRF, The European Synchrotron, CS40220, Grenoble 38043, France

**Keywords:** *in situ* studies, Y zeolite, principal component analysis, enthalpy of adsorption, entropy of adsorption

## Abstract

The increasing efficiency of detectors and brightness of X-rays in both laboratory and large-scale facilities allow the collection of full single-crystal X-ray data sets within minutes. The analysis of these ‘crystallographic big data’ requires new tools and approaches. To answer these needs, the use of principal component analysis (PCA) is proposed to improve the efficiency and speed of the analysis. Potentialities and limitations of PCA were investigated using single-crystal X-ray diffraction (XRD) data collected *in situ* on Y zeolite, in which CO_2_, acting as an active species, is thermally adsorbed while cooling from 300 to 200 K. For the first time, thanks to the high sensitivity of single-crystal XRD, it was possible to determine the sites where CO_2_ is adsorbed, the increase in their occupancy while the temperature is decreased, and the correlated motion of active species, *i.e.* CO_2_, H_2_O and Na^+^. PCA allowed identification and elimination of problematic data sets, and better understanding of the trends of the occupancies of CO_2_, Na^+^ and water. The quality of the data allowed for the first time calculation of the enthalpy (Δ*H*) and entropy (Δ*S*) of the CO_2_ adsorption by applying the van ’t Hoff equation to *in situ* single-crystal data. The calculation of thermodynamic values was carried out by both traditional and PCA-based approaches, producing comparable results. The obtained Δ*H* value is significant and involves systems (CO_2_ and Y zeolite) with no toxicity, superb stability and chemical inertness. Such features, coupled with the absence of carbonate formation and framework inertness upon adsorption, were demonstrated for the bulk crystal by the single-crystal experiment, and suggest that the phenomenon can be easily reversed for a large number of cycles, with CO_2_ released on demand. The main advantages of PCA-assisted analysis reside in its speed and in the possibility of it being applied directly to raw data, possibly as an ‘online’ data-quality test during data collection, without any *a priori* knowledge of the crystal structure.

## Abstract 2

*In situ* single-crystal X-ray diffraction data were used to unravel the structural dynamics and enthalpy and entropy of adsorption of CO_2_ into Y zeolite. A principal-component-analysis- (PCA) based approach is applied in an innovative way to single-crystal X-ray diffraction data analysis, allowing one to selectively detect the information from the subset of active atoms. The potential of and limitations of PCA in single-crystal diffraction are discussed.

## 1. Introduction   

### 1.1. On the need for novel, efficient and fast approaches to single-crystal X-ray diffraction data analysis   

The availability of faster detectors and brighter laboratory and large-scale facility X-ray sources allows the collection of a full single-crystal X-ray diffraction (XRD) data set within seconds (or even less) to minutes and the automatic collection of data sets (especially in the macromolecular crystallography field) increases dramatically the number of collected crystal structure data sets. *In situ* single-crystal diffraction will become far more common in the next few years, just as *in situ* powder diffraction has become more widespread in recent decades. Moreover, the novel approach named ‘serial crystallography’ at X-ray free-electron laser (XFEL) facilities will further increase the amount of data and crystal structure data sets to be analysed (Standfuss & Spence, 2017 ▸ and references therein). A similar increase in the amount of data is also observed at synchrotron facilities, for macromolecular systems, where the approach of serial crystallography is becoming routine (Weinert *et al.*, 2017 ▸). *In situ* single-crystal structural studies can be carried out to examine simple and slow kinetics (minutes to hours, or while studying systems at equilibrium as in the present work), also with laboratory facilities (Vergentev *et al.*, 2015 ▸). Taking into account these considerations, the number of collected data sets will soon greatly override the capacity of structure refinement analysis by the traditional manual approach, where the human operator analyses one crystal data set at a time. It is thus evident that there is an increasing need for efficient tools to manage and analyse large single-crystal data sets coming from *in situ* and/or serial crystallography.

### 1.2. PCA as a possible complementary tool in XRD data analysis   

In the present article, we propose a principal-component-analysis- (PCA) based approach which is able to answer the issues raised in Section 1.1. PCA is a blind dimensionality-reduction method used to obtain an effective representation of the system under investigation with a lower number of variables (called principal components, PCs) than in the original case (Massart *et al.*, 1998 ▸, 1988 ▸). The loadings (weights of the original variables along the PCs) and the scores (projection of the samples on the space given by the PCs) allow the identification of relationships between the variables. PCA is widely used in analytical chemistry and in the biological and ecological fields, but has rarely been applied to material science (Rajan, 2005 ▸; Rajan *et al.*, 2002 ▸). We recently demonstrated that PCA can unravel kinetic trends without a structural model when applied to X-ray powder diffraction (XRPD) data (Guccione *et al.*, 2018 ▸; Palin *et al.*, 2015 ▸, 2016 ▸) and in the present article we extend for the first time its application to single-crystal XRD data.

### 1.3. The scientific case   

As a test case, we choose a hot topic example, *i.e.* the study of the adsorption of CO_2_ into Y zeolite. The alarming data on climate change have prompted increasing efforts in developing methods for the reduction of CO_2_ in the atmosphere (Lee & Park, 2015 ▸). Nanoporous materials such as carbon molecular sieves (Lee & Park, 2015 ▸), metal–organic frameworks (MOFs) (Kim *et al.*, 2017 ▸) and zeolites (Pham *et al.*, 2014 ▸; Stevens *et al.*, 2008 ▸; Vitillo *et al.*, 2018 ▸) are promising candidates for CO_2_ capture and separation thanks to their large internal surface area and micropore volume, chemical tunability, fast adsorption/desorption kinetics and low heat of adsorption. In particular, zeolite Y is interesting for its low cost of preparation and resistance to heating and cooling cycles (Agostini *et al.*, 2010 ▸). Understanding and tailoring the features of nanoporous materials to assess their performance in CO_2_ adsorption/desorption are of paramount importance to develop efficient materials. There are reports of studies on the adsorption properties of zeolites in the literature (Sarker *et al.*, 2017 ▸). However the kinetic and/or dynamic analysis requires the application of models (Lima *et al.*, 2015 ▸), which can be influenced by the nature of the interaction, including eventual interference effects among sites, and these therefore need to be known. To gain insight into the inter­actions and structural dynamics of CO_2_ adsorption into Y zeolites, an *in situ* single-crystal XRD experiment was performed, which allowed us to locate and follow the CO_2_ atomic positions and occupancy at the different temperatures and the response of the framework and cations to the adsorption. Besides the classical *ab initio* approach of solving and refining the crystal structure from each data set taken independently, two automatic approaches were tested. At first, automatic structure refinement of the whole *in situ* data set was carried out using *TOPAS-Academic* (Coelho, 2012 ▸). Then a PCA-assisted analysis was carried out.

## 2. Methodology innovations   

PCA is applied here for the first time to the analysis of single-crystal data; it has the advantage of processing all data sets at the same time and does not require one to solve and refine the crystal structures from each data set. It is exploited here three times with two different approaches. PCA was first applied directly on experimental (case 1) and on simulated (case 2) intensities to extract the dynamic trends within the data; in these cases the variables are the Miller indices identifying the diffraction spots and the samples are the *hkl* intensities in the data sets collected at different temperatures. PCA was then applied in a different way to the occupancies obtained by a structural refinement (case 3) to facilitate the interpretation of the results from a traditional crystallographic approach; the variables in this case are the atomic species, and the PCA cases are again the data sets collected at the various temperatures.

The *in situ* single-crystal structural approach to study CO_2_ adsorption in Y zeolite is unprecedented; in the literature there are only reports of a study by XRPD (Arletti *et al.*, 2016 ▸) and a few single-crystal studies of CO_2_ adsorption on other types of zeolite performed under *ex situ* conditions (Fujiyama *et al.*, 2013 ▸; Nguyen *et al.*, 2014 ▸). Structural studies of the adsorption in Y zeolite of organic molecules can also be found in the literature (Martucci *et al.*, 2015 ▸; Gigli *et al.*, 2018 ▸). In this study traditional structural refinement (both manual and automatic) and PCA-based approaches are thus explored and the results compared to assess potentialities and limitations of PCA applied to single-crystal data. The manual structure refinement is able to locate extra-framework atoms in an accurate way after structure solution but requires a model structure and is very time consuming. The refinement of a data set of 50 structures takes many working hours and a protocol has to be defined in order to treat all the structures consistently. The automatic (batch) refinement is faster, but still needs the structure to be solved and careful checking of results and refined variables. In contrast, PCA can be executed on the full data set; it does not need structural information; and can provide information about the trends in the data in a few minutes. The trends extracted by PCA can be coupled with structural information obtained from one or more data sets to gain a better insight into their meaning (*i.e.* to link the changes to the relevant part of the structure).

Finally, the thermodynamic parameters (enthalpy and entropy of the adsorption) were calculated by the van ’t Hoff equation, extending its application from *in situ* Fourier transform infrared spectrometry (FTIR) (Garrone *et al.*, 2017 ▸; Garrone & Areán, 2005 ▸) to *in situ* XRD.

## 3. Experimental and data analysis details   

### 3.1. The *in situ* single-crystal diffraction experiment   

A suitable crystal of Y zeolite for single-crystal measurements was obtained by the hydrothermal method described in the literature (Warzywoda *et al.*, 1999 ▸; Ferchiche *et al.*, 2001 ▸) and detailed in the supporting information. The experiments were performed at the ESRF Synchrotron in Grenoble, on the Swiss–Norwegian Beam Line (SNBL; BM01A) equipped with a 2D Pilatus 2M detector. The single crystal was mounted on a 0.2 mm capillary and sealed inside a 0.3 mm capillary. The external capillary was connected to a gas line and a vacuum pump and left at 300 K under vacuum for the outgassing. One first data set, to test the sample and to be used as a reference, was collected under vacuum at 300 K. CO_2_ was injected into the capillary to a pressure of 10 kPa and the temperature was lowered to 200 K, while measuring a complete single-crystal data set (360° φ scan with an exposure time of 2 s per frame) every 2 K. Between two data sets the dead time was 2 min to allow the cryostream to reach and stabilize at the temperature and the sample to reach its equilibrium.

### 3.2. Details of the data analysis   

Data reduction was performed using *CrysAlisPro* (Rigaku, Oxford Diffraction). The data were treated at first by *SHELXL* (Sheldrick, 2015 ▸) with the *SHELXLE* interface (Hübschle *et al.*, 2011 ▸) and then by *TOPAS-Academic* (Coelho, 2012 ▸) in single-crystal diffraction batch analysis mode. Simulated *hkl* files were generated by *TOPAS* giving the desired occupancies (from linear or polynomial fitting or from the refined data) as input to the structure model and obtaining the calculated profile as output. PCA of simulated, experimental data and refined occupancy values was carried out by *RootProf* (Caliandro & Belviso, 2014 ▸). Graphics were generated using CCDC *Mercury* (Macrae *et al.*, 2008 ▸) and *Vesta* (Momma & Izumi, 2011 ▸). The 200 and 300 K refined crystal structures are given as CIFs in the supporting information. Their *checkCIF* files present a number of unavoidable *C* and *G* alerts due to the disorder of CO_2_, water and Na^+^ moieties. The 300 K CIF also contains one *B* alert due to the larger disorder observed at the higher temperature.

## 4. Dynamic structural analysis   

### 4.1. Location of extra-framework species   

Individual structural refinement of *in situ* single-crystal data was performed using *SHELX*. The data revealed that the cell parameters increased while decreasing the temperature, as CO_2_ was filling the pores (Fig. S1). The crystal structure was at first solved and refined from three data sets, collected at the temperatures 200, 254 and 300 K, revealing that not only CO_2_, but also Na^+^ ions and a residual water molecule moved noticeably during the experiment. The refinement of all the atom positions of Na^+^, CO_2_ and water is difficult to handle in a consistent way for all the data sets. Such a refinement became unstable in batch mode and the resulting atomic shifts were difficult or impossible to interpret. A structural model was then built from the *SHELX* refinement of one data set out of five (every 10 K), considering all positions of CO_2_, water and Na^+^ atoms found from manual structure solution. Concerning CO_2_ conformation, it is generally considered that CO_2_ in zeolites can be found as a linear molecule, even if density functional theory (DFT) calculations (Wong-Ng *et al.*, 2013 ▸) suggest that a bending of CO_2_ (with an OCO angle of about 150°) requires a limited energy cost. On the other hand, the partial conversion of CO_2_ to carbonate (OCO bond of 120°) may result in an electron density interpretable only by a bent CO_2_ (Wong-Ng *et al.*, 2013 ▸). For these reasons, the initial refinement of CO_2_ was carried out without constraints on the OCO bond angle to verify CO_2_ linearity.

**Figure 1:**
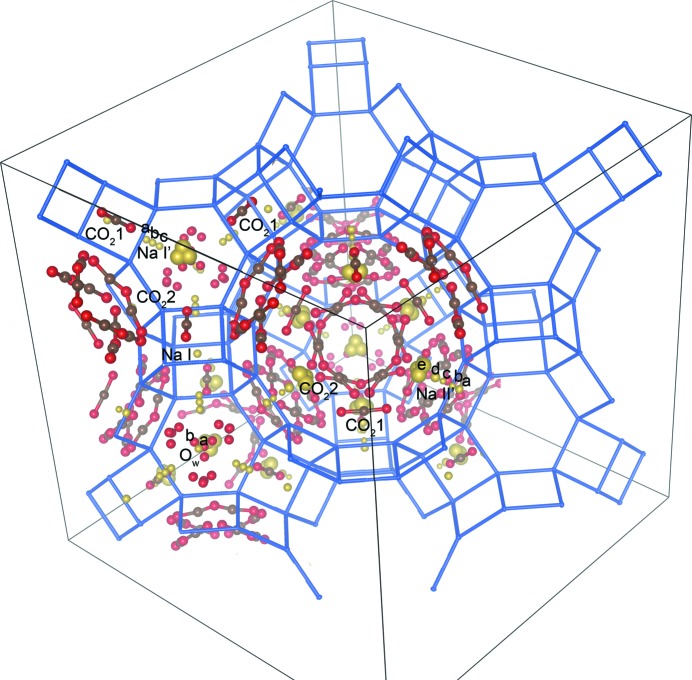
Zeolite Y structure highlighting CO_2_ and Na^+^ sites (Si = blue; Na = yellow; O = red; C = brown).

Looking to the experimental electron density and to the unconstrained refined position, carbonate species are unlikely; moreover, any attempt at locating and refining bent CO_2_ molecules or carbonate species failed or gave increased agreement factors and/or higher atomic displacement parameter (ADP) values. Only linear CO_2_ molecules were then used in all the manual and sequential refinements. This evidence confirms that carbonate species are present in X zeolites only (Arletti *et al.*, 2016 ▸; Pirngruber *et al.*, 2010 ▸; Busca, 2017 ▸; Wong-Ng *et al.*, 2013 ▸) and are unlikely in Y zeolite. The positions of Na atoms were treated as disordered sites, *i.e.* by fixing their coordinates and refining their occupancies (Fig. 1 ▸).

### 4.2. Dynamic structural refinements   

The refinement was performed manually on one data set out of five. This approach allowed tracking of the movements as a function of a single variable (occupancy) instead of three (*x*, *y*, *z*), facilitating the interpretation of the results (Fig. 1 ▸ and Fig. S4 in the supporting information). The agreement factors (Figs. S2 and S3) confirmed the quality of the collected data and the goodness of fit of the refinements. A sequential batch refinement of all the patterns was then performed using *TOPAS* in single-crystal mode. The refinement conditions used in *TOPAS* were set to be consistent, as much as possible, in terms of fixed parameters and constraints with the *SHELX* refinement. The thermal factors were refined at 254 K and kept fixed in the automatic sequential refinement. The occupancy of the CO_2_ positions was refined constraining all atoms of the same molecule to have the same occupancy.

**Figure 2:**
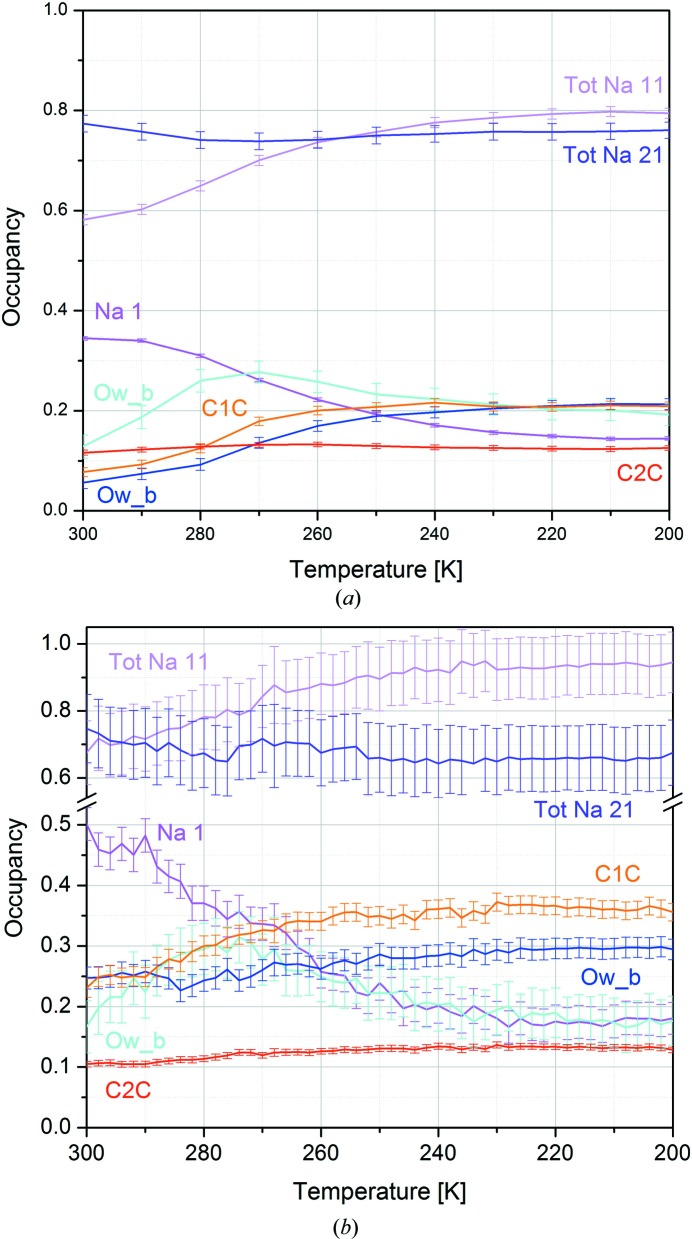
Occupancy of CO_2_ (C1*C* and C2*C* atoms representing the two CO_2_ moieties), water (O*w*_*a* and O*w*_*b*) and Na^+^ during the thermal treatment from manual (*a*) and sequential (*b*) refinements.

The total occupancy of Na atoms was also constrained after the first refinements. In this way, a stable refinement was obtained. Despite the different number of samples and the different software used, the manual and automatic approaches gave very similar results (Tables S1 and S2), except for the Na^+^ occupancy, which is found to be systematically larger by *TOPAS* (Fig. 2 ▸ *b*). The analysis of the refined structures revealed at first a change of the Na distribution among the different positions upon CO_2_ adsorption when the temperature changes from 300 to 200 K. When the CO_2_ occupancy increases, Na^+^ ions in the sites I′ (from now on Na11) and II′ (from now on Na21) far from the centre of the sodalite cage and close to CO_2_ increase their occupancy (Figs. 2 ▸ *a*, 2 ▸ *b*). At the same time, the depletion of the Na^+^ position at the centre of the hexagonal prism (site Na I, from now on Na1) is observed. This is in line with the molecular dynamics simulation performed by Plant *et al.* (2006 ▸). The water molecule near the centre of the sodalite cage moves towards the Na^+^ ions from position *a* (O*w*_*a* at higher symmetry) to position *b* (O*w*_*b*) at lower symmetry. The shift of the atoms, represented by occupancy variation between the positions, is highlighted by the animation provided as supporting information.

### 4.3. PCA as a complementary tool for analysis of *in situ* single-crystal diffraction data   

PCA was applied in three different modes, *i.e.* using directly *hkl* intensities or the refined occupancy values as the input data matrix. Cases 1, 2 and 3 as defined at the beginning of Section 2 are described in the next two subsections.

#### 4.3.1. PCA directly applied to X-ray simulated and experimental diffraction data   

**Figure 3:**
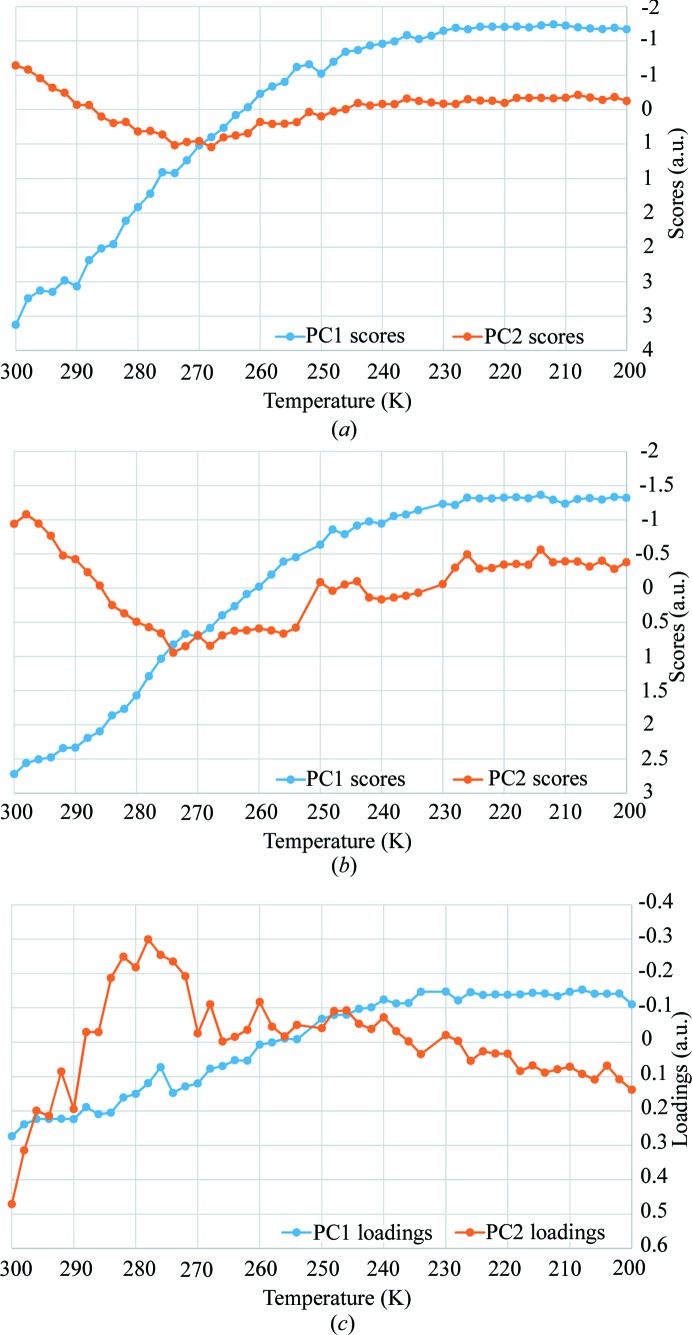
Scores of PC1 and PC2 from PCA applied to case 1, *i.e.* data from simulation 5 (*a*), case 2, *i.e.* experimental data (*b*), and case 3, the loadings obtained after the PCA giving as input the occupancies from sequential refinement (*c*). The scatter plot of these analyses is reported in the supporting information (Figs. S12–S14); the analysis was limited to the first two PCs, able to explain 95 and 97% of the variance in experimental and simulated data, respectively.

Besides the above-described manual (*SHELX*) and automatic (*TOPAS* TA) traditional approaches of structure refinement and analysis, the efficiency of multivariate methods in following structural changes occurring during *in situ* experiments (Palin *et al.*, 2015 ▸, 2016 ▸) was exploited by applying PCA to these single-crystal data. As a preliminary test, since the application of PCA to *in situ* single-crystal XRD data is unprecedented, a simulated experiment was carried out and analysed by PCA (case 1 as defined in Section 2) as described for polycrystalline samples by Palin *et al.* (2015 ▸, 2016 ▸). Simulated *hkl* data were generated starting from the refined structure and the occupancies of a subset of atoms varied. This approach allowed us to test PCA starting from an ideal data set and introducing complexity step by step, to facilitate the comprehension and interpretation of the results. Several simulations were performed as summarized in Table SI-1: in simulations 1, 2 and 3, only the occupancies of selected species were varied with simple linear trends, in simulation 4 a polynomial trend was used, while in simulation 5 all occupancy variables were varied according to the occupancies obtained from refinement on real data. The cell parameter was kept fixed in all simulations. The PCA results are shown in the supporting information (Figs. S5–S8). The PCA scores of simulation 5 (Table SI-1) are shown in Fig. 3 ▸(*a*), since this simulation is the most similar to the real experiment. In the simplest cases, PCA scores are able to reproduce the introduced CO_2_ occupancy variations; we call the subset of varying atoms ‘active species’.

As a first consideration, it must be pointed out that PCA can be used to estimate the quality of the data when tracking the changes occurring during an *in situ* experiment. In fact, as widely discussed by Palin *et al.* (2015 ▸) for powder diffraction data, the PC1 versus PC2 scores must show a parabolic behaviour if an occupancy variation is observed within a data set. This is clearly also visible in simulated data (Figs. S5–S8C) for the single-crystal case. This trend is still observable in the more complex simulation, close to real data (Fig. S12), and is also observed in the real data (Fig. S13), even if the experimental errors induce deviations from the theoretical behaviour. The presence of such parabolic trends ensures a good quality of the data and a correct recovery of the dynamic trends within the *in situ* XRD data set. In these ideal and simple situations, PC1 scores unravel from raw *hkl* intensities the dynamic trend of the active species within the simulated or real experiment, *i.e.* the response of the sample to the applied stimulus.

In more complex cases than the real one investigated in this study, PCs mix contributions from different active species, having similar trends of occupancy variations. When PCA was applied to real *in situ* data (case 1 as defined in Section 2), a discontinuity in the data trend as a function of time was found (Fig. S9). It is captured by PC1 scores, which match the scale parameter obtained by the *TOPAS* batch refinement, and can be ascribed to a synchrotron refill which occurred during the measurements. This issue is automatically handled by *SHELX* and *TOPAS* through the scale factor, but should not be overlooked when dealing with raw data by a blind approach such as PCA. In this case, the maximum variance of the input data set is in fact the parameter known in structure refinement as the scale factor. PCA is not *a priori* able to recognize a scale effect from genuine structural variations. To overcome the problem, the pre-processing features of *RootProf* (normalization) were therefore applied to normalize the data. PCA then showed some outliers (Fig. S10), which were therefore investigated and excluded from the analyses. The data set ‘232 K’ was collected during the refill; therefore there are discrepancies in the intensities of the peaks. The data set at 252 K also showed some anomalies and a careful inspection of the data reduction statistics revealed problems during data collection. In fact, the ‘absscale’ parameter of this data set (Fig. S11) has a very unusual trend (the expected behaviour is a nearly flat line) with a bump in the first frames and two very negative values. These two examples highlight the potentialities of PCA in data quality control during the experiment, especially in those cases where a large number of data sets (hundreds or thousands) are collected thanks to the improved time resolution. The plots of PC1 and PC2 scores on the pre-processed normalized data are given, after excluding the two outlier data sets (232 and 252 K), in Fig. 3 ▸(*b*) and show very similar trends compared with those from simulated data [Fig. 3 ▸(*a*)]. By comparing the trends of occupancies shown in Fig. 2 ▸ and PCA results in Fig. 3 ▸, PC1 shows a trend similar to that of the occupancies of CO_2_, Na 11 and O*w*_*b*, while PC2 resembles the trend of O*w*_*a*. Since PCA explains the variance contained in the data set, the signs of the scores and loadings of each PC are arbitrary. They can be flipped if the sign of both scores and loadings is reversed at the same time. It must be noticed that data points in Figs. 2 ▸(*a*) and 2 ▸(*b*) show different scattering between adjacent data sets. This is due to the different sampling in manual (one data set used out of five with 10 K between adjacent data sets) and batch refinement and PCA (all data sets used with 2 K data sets) analyses. The change in the structure between two adjacent data sets is smaller than the error in the batch refinement and PCA [Figs. 2 ▸(*b*) and 3 ▸(*b*), respectively] while it is larger than the error in the manual refinement [Fig. 2(*a*)].

#### 4.3.2. PCA applied to occupancy values from the structural refinement   

**Figure 4:**
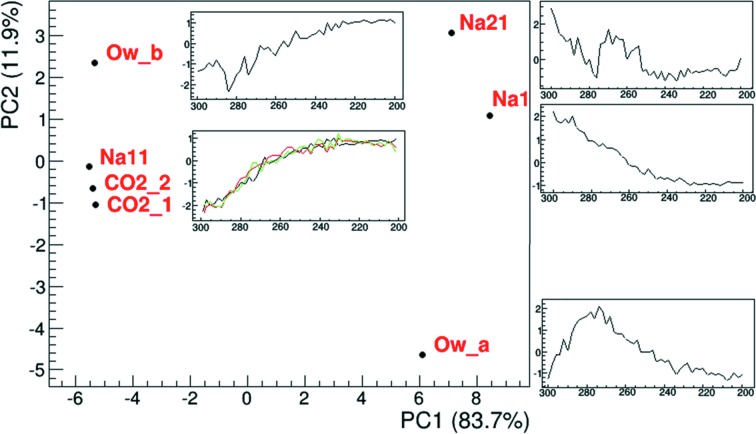
Plot of PC2 versus PC1 scores from PCA applied to occupancy values obtained from the sequential refinement. Each point in the plot corresponds to the site indicated by the label. The insets show the normalized trends of occupancies (found by the automatic *TOPAS* TA refinement given as input in PCA case 3) next to the related point in the plot, grouped according to the score plot.

PCA was then used with a different approach, defined as case 3 in Section 2 [Fig. 3 ▸(*c*), Fig. 4 ▸]. PCA was performed using the atomic occupancies (instead of *hkl* intensities as in cases 1 and 2) versus temperatures as input. This allowed us to compare known structural features obtained by the refinement procedure, and to explain the correspondence between PCs and active species. In this way, it is possible to directly associate the species to PCA trends. A pre-processing based on standard normal variate transformation (Caliandro & Belviso, 2014 ▸) has been applied to the refined occupancy values, to account for different absolute occupancy values.

By comparing the loading obtained by PCA performed on refined occupancies [Fig. 3 ▸(*c*)] with the scores obtained by PCA performed on raw data [Fig. 3 ▸(*b*)], we can see that the trends in this case are also very similar, thus demonstrating that PCA can be applied directly to raw data. It must be noted that in cases 1 and 2 the scores represent the trend of the changes, while in case 3 the loadings describe the trends. The difference depends on the fact that, in cases 1 and 2, the *hkl* intensities are given as input of the PCA, while in case 3 the occupancy trends (inset of Fig. 4 ▸) are given as input. In fact, by definition in the PCA procedures, the loadings are the coefficient defining the new coordinates and they therefore describe the input variables in the new basis of PC1, PC2, …, PC*x*. In other words, they are the projection of the input variable in the new basis. The number of PCs to be considered is chosen to explain at least 90–95% of the variance of the original data set. The scores describe the variance of the input variable. Therefore, giving *hkl* intensities as input (cases 1 and 2) the scores describe the structural variation within the experiment, contained in *hkl* variations, and we demonstrated that they represent the ‘occupancy trends’ [Figs. 3 ▸(*a*), 3 ▸(*b*)]. Giving occupancy trends [Fig. 2 ▸(*b*)] as input (case 3), the loadings give back the trend described by the new basis (PC1 and PC2 in Fig. 3 ▸) while the scores (Fig. 4 ▸) represent the classification of the occupancy variations, *i.e.* they tell us which atoms vary with similar trends.

However, assigning a structural interpretation to the trends extracted by the PCA from raw data requires a traditional refinement approach to find the correspondence between the direct and reciprocal space. The necessity of solving and refining the structure can be avoided if a simple and evident change occurs, *e.g.* water elimination in porous or layered solids. In the present case study, assigning the trends observed by PCA in *hkl* intensities to chemical behaviour is impossible without a structural refinement. However, PCA can also be used as a tool to analyse trends in the refined occupancies [Fig. 2 ▸(*b*)], if these are given as input. In this case, the plot of the scores of each PC (Fig. S15) indicates which atomic species has the larger influence on that PC while the PC1 versus PC2 score scatter plot (Fig. 4 ▸) gives at first glance the similarities between the trends of different atomic species. The analysis of the scores of the PCA applied to occupancies refined by *TOPAS* PC1 versus PC2 loading scatter plot can thus give an overview of the variations and features of the trends [Fig. 3 ▸(*c*)] as a function of temperature, and connect them to a particular atom. In addition, in this case PCA is very efficient in classifying the trends coming from structural refinement. The PC2 versus PC1 score plot (Fig. 4 ▸) shows data points representing the active species positioned according to their coordinate in the new PCA basis and, next to them, the corresponding refined occupancy profiles as a function of temperature. A close position of two points in Fig. 4 ▸ implies that the corresponding refined occupancies behave in a similar manner. The closer the points, the more similar the behaviours. PCA was repeated giving as input the occupancies of each single position. This analysis highlights the peculiar trends of Na21 positions, being rather scattered in Fig. S16. This implies that each Na21 position behaves differently from the other ones, because of the different interaction with CO_2_ molecules.

Of course, the information is not different from visual observation of the trends [*cf*. Fig. 2 ▸(*b*) and the insets of Fig. 4 ▸], but has the advantage of being automatic, very fast, unbiased and quantitative, with respect to the traditional comparative approaches. Moreover, the method is multivariate in the sense that trends of many variables are identified and compared at the same time. The ‘manual comparison’ method requires an increasing amount of time comparing 10 to 100 trends and it is impossible with a larger number of different trends (consider, for instance, tracking amino-acid changes in an *in situ* protein XRD study). The PCA method performs the analysis reported in Fig. 4 ▸ in seconds to minutes even if more than 1000 occupancy (or other structural parameter) trends are observed.

#### 4.3.3. Chemical considerations on observed occupancy trends   

It can be observed that atoms in Fig. 4 ▸ are divided into two groups. Therefore, PC1 is able to discriminate the species that increase their occupancies with temperature (Na1, Na21 and O*w*_*a*) from those having the opposite behaviour (*i.e.* the CO_2_ moieties, Na11 and O*w*_*b*) (*cf*. PC1 trend in Figs. 3 ▸ and 4 ▸). PC2 instead discriminates occupancies according to the shape of the occupancy variations, and distinguishes the monotonic behaviour, followed by Na atoms and the CO_2_ moieties, from the more complex trend of water atoms (*cf*. PC2 trend in Figs. 3 ▸ and 4 ▸), which show an inversion point at about 270 K when CO_2_ adsorption reaches an inflection point. The apparent increase in occupancy of water sites with temperature decrease (PC2 plot in Fig. 3 ▸) could be due to the reduction of thermal motion and to the fact that a little bit of water is desorbed shifting back from position *a* (in a special position) to position *b* (towards the side of the cage) upon CO_2_ adsorption (Fig. 1 ▸). This analysis explains why PCA finds two main trends in the raw data: (i) the monotonic occupancy variations of most atoms, both increasing and decreasing with temperature, captured by PC1 (Fig. 3 ▸), and (ii) the peculiar behaviour of water molecules, which deviates from the monotonic trend for temperatures higher than 270 K, captured by the second PC (PC2) (Fig. 3 ▸).

## 5. Thermodynamic calculations   

The calculation of the enthalpy and entropy of CO_2_ adsorption was then performed, extending to *in situ* XRD the approach proposed by Garrone *et al.* for *in situ* FT–IR data (Garrone *et al.*, 2017 ▸; Garrone & Areán, 2005 ▸), using the refined occupancies as an indicator of CO_2_ adsorption. The calculation of these parameters is possible because the timing of the experiment, described in Section 3, allowed collection of each data set at equilibrium, without evident evolution of the system during individual measurements. This is confirmed and demonstrated by the good values of *R*_int_ and *R*(sigma) [see Fig. S2 (top)] of each data set (they would be higher and more irregular in the temperature range where adsorption occurs if the system were evolving within the same data set). Moreover the 2D raw data frames collected at the beginning and at the end of the data set would have given many inconsistent intensities for equivalent reflections [Fig. S2 (bottom)] if the system were evolving.

**Figure 5:**
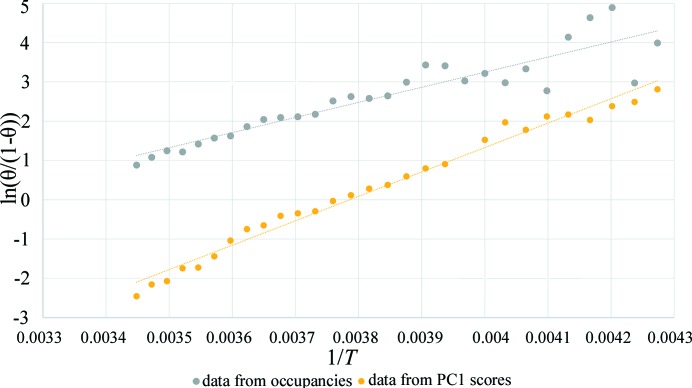
Plot and linear regression of the linearized van ’t Hoff equation. In the grey plot, θ is calculated as occCO_2_/occCO_2max_. *y* = 3842.6*x* − 12.123, *R* ^2^ = 0.8486. In the yellow plot, θ is calculated as PC1/PC1_max_, using PC1 scores from the analysis of real data. *y* = 6212.3*x* − 23.52, *R* ^2^ = 0.983.

Therefore, the van ’t Hoff equation can be exploited to obtain the thermodynamic parameters of the process. The equation in its linearized form [equation (2) in the supporting information] was used to build the plot in Fig. 5 ▸. The total CO_2_ occupancies from Rietveld refinement were used to calculate θ, *i.e.* the fraction of occupied adsorption sites. The slope and the intercept, obtained from linear regression, together with their standard deviation, allowed us to calculate [see equation (2) in the supporting information] the values of Δ*H* = −32 ± 3 kJ mol^−1^ and Δ*S* = −100 ± 10 J Kmol^−1^, using the data from 234 to 290 K, thus eliminating the low and high temperatures where occupancies are asymptotically constant. This range was selected by visual inspection (Fig. 3 ▸) and further confirmed by the best *R* ^2^ value in the interpolation of the van ’t Hoff equation (Fig. 5 ▸). The values are in agreement with data reported for CO_2_ in MFI zeolite (Armandi *et al.*, 2009 ▸). The corresponding plot obtained using the PC1 scores as CO_2_ amount estimator is reported in Fig. 5 ▸ (after switching the sign according to *a priori* knowledge that adsorption is exothermic) and gives an approximate value of Δ*H* = −52 ± 1 kJ mol^−1^ and Δ*S* = −196 ± 6 J Kmol^−1^. The difference compared with values obtained by the occupancy data from refinement is important, but obtaining the same order of magnitude with a totally blind and much faster method, not requiring *a priori* information on the crystal structure, is noteworthy. PCA can thus be a very efficient tool to obtain thermodynamic parameters by PCA applied on raw data as soon as they are collected, useful as a preliminary investigation and data-quality check. In other words, when thermodynamic calculation carried out by PCA during the experiment gives unlikely results, possible problems in experiment execution can be inferred, found and solved.

## 6. Conclusions and perspectives   

### 6.1. Structural and thermodynamic results   

An *in situ* single-crystal XRD experiment, followed by an automatic and PCA-assisted data analysis, allowed an unprecedented clear and high-resolution view of structural changes during CO_2_ adsorption into Y zeolite. Even if the single crystals suffer from reduced diffusion and a lack of statistical significance with respect to the typical nanometric or micrometric polycrystalline samples, the structural dynamics disclosed during cooling in a CO_2_ atmosphere give a realistic starting point for interpreting the events occurring in a powder sample. Summarizing the results on the scientific case, Na atoms are ‘pulled’ towards CO_2_ during its adsorption into the zeolite channels. Water molecules are, at the same time, moved away from the centre of the cage. Changes in every site can be tracked singularly by both manual and automatic traditional crystal structure refinements. The enthalpy and entropy of CO_2_ adsorption were calculated as Δ*H* = −32 kJ mol^−1^ and Δ*S* = −100 J Kmol^−1^, respectively. These values are consistent with those calculated from FT–IR data by Armandi *et al.* (2009 ▸), thus demonstrating the possibility of obtaining thermodynamic data from *in situ* single-crystal diffraction. The enthalpy value is significant and involves systems (CO_2_ and Y zeolite) with no toxicity, superb stability and chemical inertness. The XRD experiment indicated the absence of carbonate formation and that zeolite changes are limited to Na-ion movement, with the framework unchanged before and after adsorption. The CO_2_ adsorption phenomenon can thus be easily reversed, with CO_2_ released on demand, with a heat recovery. The system can therefore be considered both as an efficient CO_2_ and a heat tank, available on demand at temperatures close to room temperature.

### 6.2. Methodological innovations: potentialities and limitations of PCA in single-crystal XRD   

The potentialities and limitations of automatic refinement by *TOPAS* and of the PCA-assisted analysis were tested on an *in situ* single-crystal data set. Automatic analysis by *TOPAS* was faster and was applied to all 50 data sets (instead of the ten manually refined data sets where only one out of five data sets is analysed). Automatic refinement was stable after some manual data set analyses were performed to optimize the automatic refinement procedure and determine the range of variation of parameters, mainly occupancies and ADPs in a gas adsorption experiment. PCA was used in two different ways. When applied to raw *hkl* data, it was able at first to identify problematic data sets and then to unravel the dynamic trends in the data without the knowledge of the structure and within minutes after data collection finished (we can infer for future experiments that this may also be possible with partial data sets during data collection). The results are similar to those obtained by more time-consuming traditional manual and automatic approaches (see Figs. 2 ▸ and 3 ▸). PCA can thus act as a diagnostic tool if performed during or soon after the measurements to highlight problems in the measurement related to the experimental setup and to data collection. This was shown by the data collection/reduction issues highlighted by the first analyses (Figs. S9–S11). The approach can be very useful to determine dynamic or kinetic trends in systems whose crystal structure is disordered in a portion of the experiment (maybe across a transition) so that the traditional refinement becomes impossible. PCA was in fact able to describe all the complex events that involve different atoms in the structure, grouping the atom movements by their ‘direction’ and ‘timing’, giving the overall kinetic information if performed on the raw data. PCA was then used in a different way to obtain insight into relationships between atom occupancy changes during CO_2_ adsorption. In this case, PCA was performed using the results from the structure refinement as the input data matrix. In particular, CO_2_, Na^+^ and water occupancy changes were analysed by PCA and the common trends obtained. PCA clearly indicates which atoms change their occupancies in a synchronized way. This information was obtained from the plots shown in Fig. 4 ▸. Finally, the possibility to exploit an *in situ* single-crystal experiment to obtain thermodynamic data by a structure-free PCA-based approach was also demonstrated. PCA was able to estimate, with a large discrepancy with respect to the traditional approach, the enthalpy and entropy of adsorption, but the trend was obtained and the order of magnitude investigated. The main limitation in this case is the difficulty of correctly scaling the data using the blind approach of PCA. A correct scaling of the data is one of the more challenging and intriguing aspects of PCA application to XRD data.

### 6.3. Perspective of PCA usage in single-crystal diffraction   

The main limitation of the PCA-based blind approach is attributing a chemical/structural sense to the investigated data, and, in this case, the traditional (manual or automatic) approaches are superior. However, PCA can be very useful to identify the subset of the collected data set that is more interesting for data analysis, *e.g.* those data sets where the dynamic effect starts, reaches the maximum speed and finishes. PCA can also be very efficient in highlighting very small dynamic effects, not evident by traditional visual analysis, and also in very large data sets. A future can be envisaged in which, after collecting hundreds or thousands of single-crystal data sets within a single (*in situ* or serial crystallography) experiment, PCA can be used to check data quality, identify problematic data sets and diminish the number of data sets to be refined manually by traditional structure-based approaches. In this way, a huge efficiency improvement will be obtained, with respect to the alternatives of refining manually all (hundreds or thousands) data sets or refining a subset of data sets selected by arbitrary methods.

## Supplementary Material

Crystal structure: contains datablock(s) global, 200K, 300K. DOI: 10.1107/S2053273318017618/sc5122sup1.cif

Animated gif showing CO2 motion at 200 and 300 K. DOI: 10.1107/S2053273318017618/sc5122sup2.gif

Other supporting information. DOI: 10.1107/S2053273318017618/sc5122sup3.pdf

CCDC reference: 1884781
