# Supplementary figures and images for: CO2 adsorption in Y zeolite: a structural and dynamic view by a novel principal-component-analysis-assisted in situ single-crystal X-ray diffraction experiment
Source: Acta Crystallogr A Found Adv. 2019 Feb 6;75(Pt 2):214–22. doi: 10.1107/S2053273318017618 (PMC6396398; doi:10.1107/S2053273318017618)

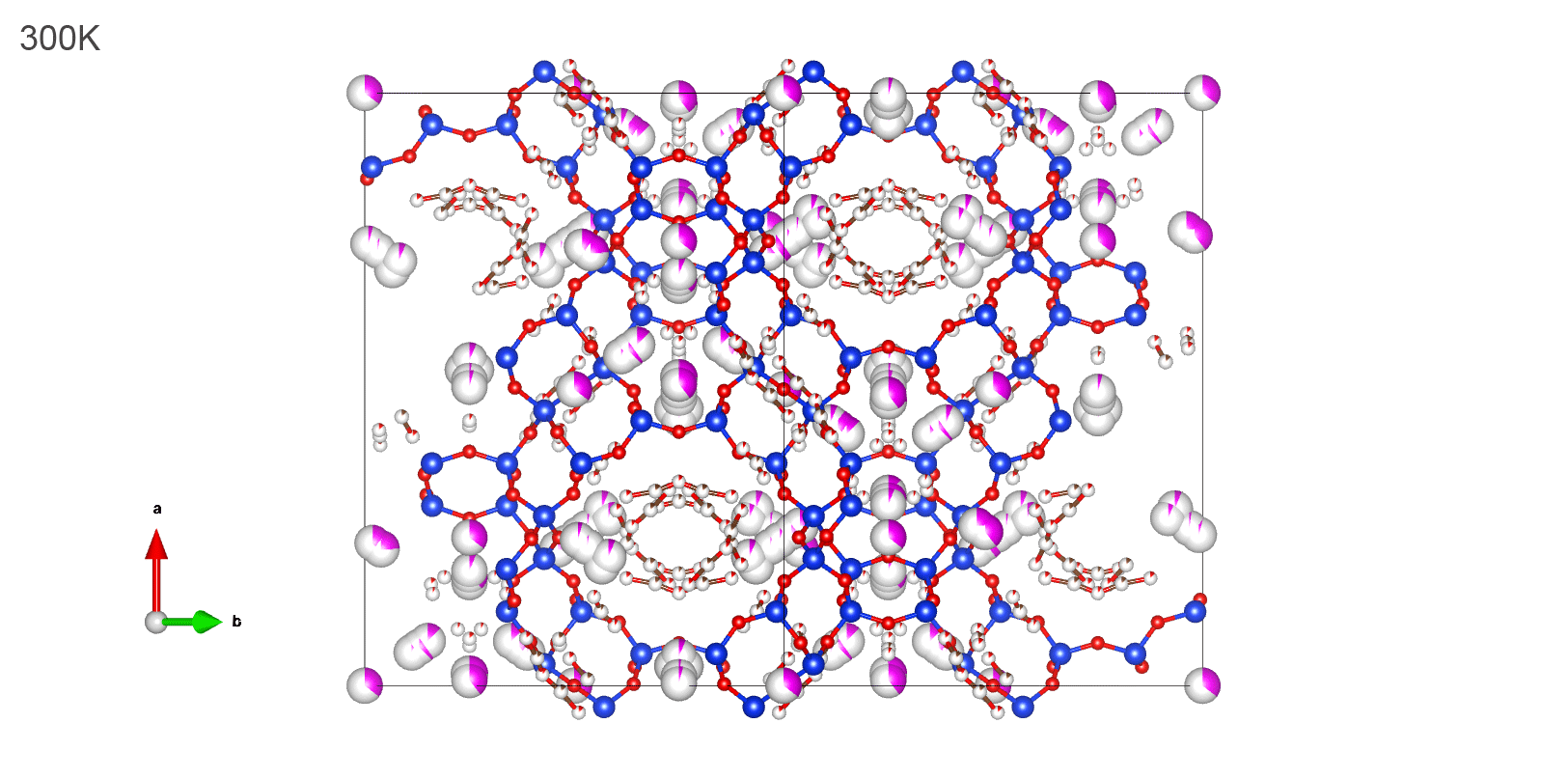

Supplement: Supplementary file 2 [file a-75-00214-sup2.gif]
