# Supplementary material for: CO2 adsorption in Y zeolite: a structural and dynamic view by a novel principal-component-analysis-assisted in situ single-crystal X-ray diffraction experiment
Source: Acta Crystallogr A Found Adv. 2019 Feb 6;75(Pt 2):214–22. doi: 10.1107/S2053273318017618 (PMC6396398; doi:10.1107/S2053273318017618)
Supplement: Supplementary file 3 [file a-75-00214-sup3.pdf]

Acta Cryst A FOUNDATIONS ADVANCES

Volume 75 (2019)

Supporting information for article:

CO2 adsorption in Y zeolite: a structural and dynamic view by a novel PCA-assisted *in situ* single crystal XRD experiment

Eleonora Conterosito, Luca Palin, Rocco Caliandro, Wouter van Beek, Dmitry Chernyshov and Marco Milanesio

S1. Preparation of zeolite Y single crystals

Two batches of SOL were prepared using different amounts of TEA.

a) 4.76Na_2O : 1.0Al_2O_3 :3.5 SiO_2 :454 H_2O: 8 TEA

b) 4.76Na_2O : 1.0Al_2O_3 :3.5 SiO_2 :454 H_2O: 5 TEA

The first solution was prepared adding 6.468g di $NaAlO_2$ to 170ml of hot NaOH (2M) solution. Then 15.19g (a) or 24.30g (b) of TEA were added to 100g of the solution. In a second becker the solution was prepared adding 0.58g of fumed SiO_2 in 10ml of distilled water. The solutions were aged one night then the first solution was filtered and added to the second. Each batch was divided into two sealed bottles. For each batch one bottle was put in oven @75°C and another @100°C.

Batch (b) was left in the oven for 8 days while batch (a) was left for 13 days. In all the bottles, clear octahedral crystals were found at the bottom of the flask. The larger crystals, of about 50 µm, were found in batch (a)@100°C while in batch (b)@100°C the crystals were of about 30 µm in size. In both batches left @75°C the crystals were less in number and less regular. No evident signs of twinning were present and the crystals formed from (a)@100°C and (b)@100°C are approximately all of the same size. For the measurements one crystal from batch (a)@100°C was chosen.

S2. X-ray diffraction data analysis of zeolite Y single crystals

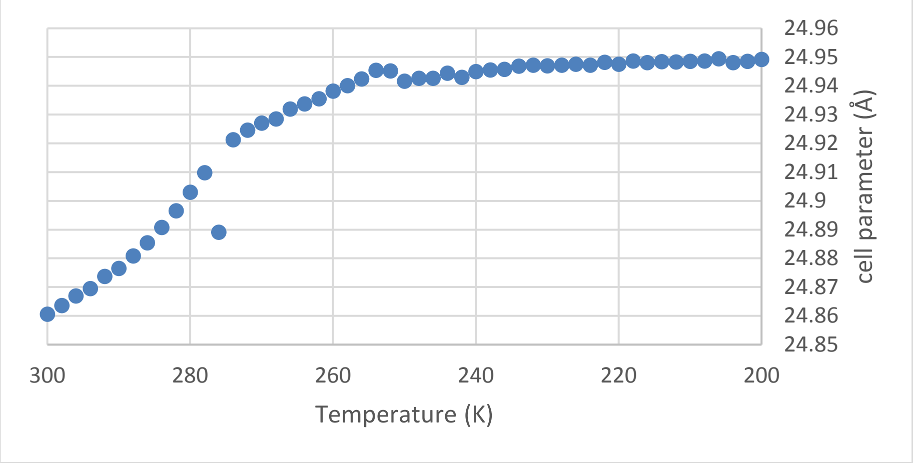

Figure S1 Cell parameter vs. temperature of Zeolite Y measured from 300 to 200 K.

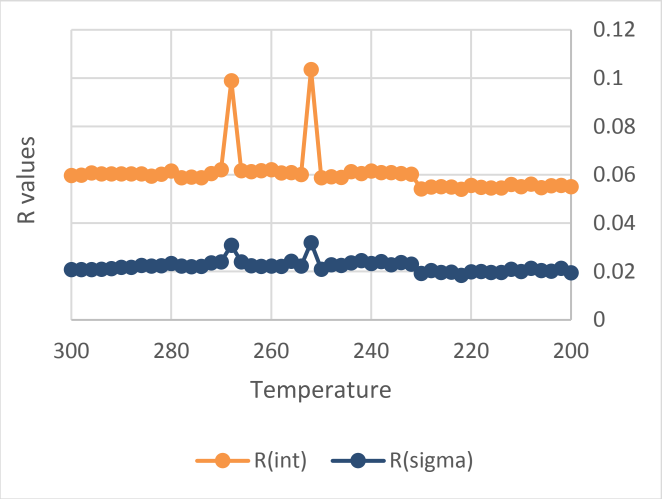

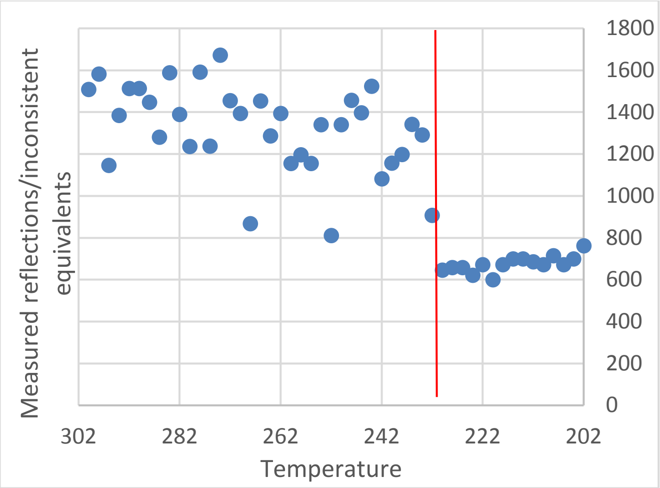

Figure S2 Plot of R(int) and R(sigma) values for each dataset in the collection (top). Reflections collected / inconsistent equivalent ratio for each dataset (bottom). The red line marks the synchrotron refill.

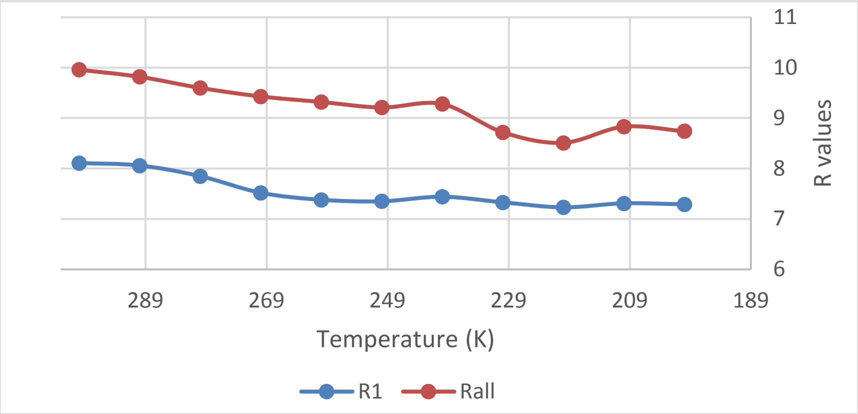

Figure S3 R value vs. temperature (K) obtained after refinement with SHELX.

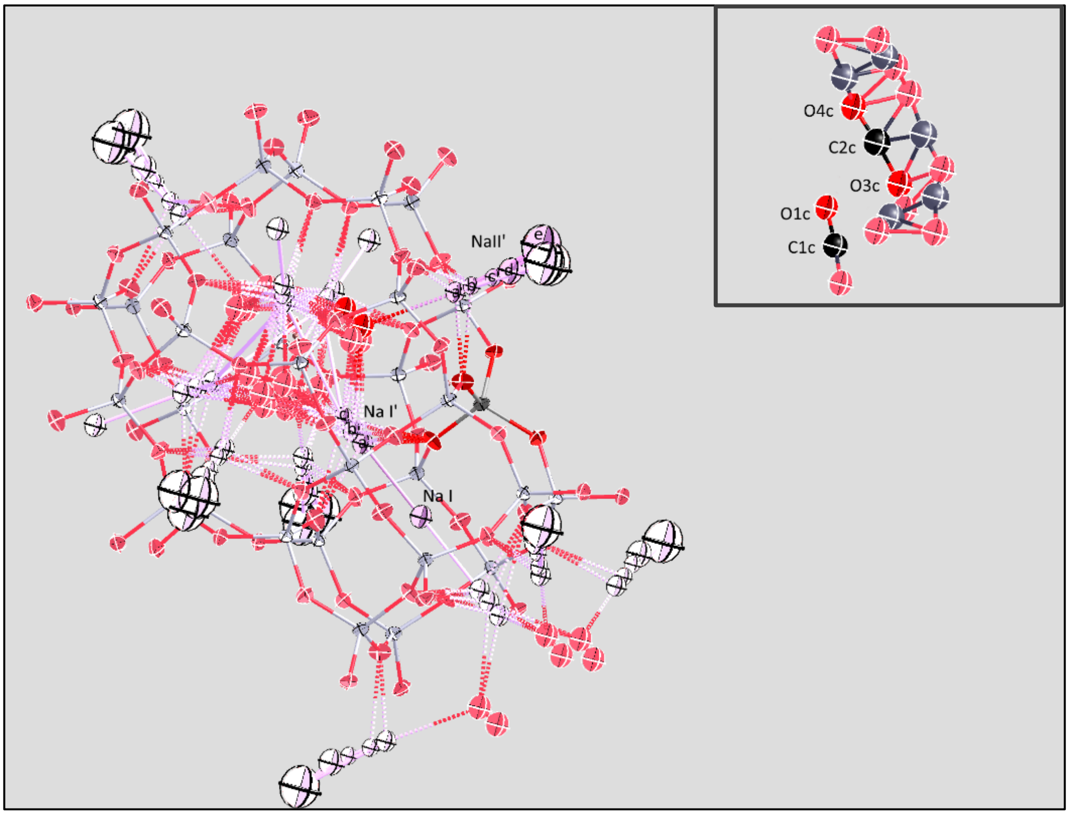

Figure S4 Structure of Zeolite Y with CO_2 and Na atoms labeled. The name of Na atom sites traditionally used in the literature are identified by numbers for sake of clarity (NaI becomes Na1, NaI' becomes Na11 and NaII' becomes Na21) and the different positions assumed during the temperature ramp are indicated by letters from **a** to **c** for position I' (Na11a is the nearest to Na1) and from **a** to **e** for position II'. Detail of the CO_2 in the inset. CO_2 is adsorbed in two positions in the large channel of the zeolite, in particular one CO_2 molecule is coordinated to Na21 (NaII'), the other CO_2 position is more disordered.

S3. PCA analysis of in situ X-ray diffraction data analysis of zeolite Y

S4. Table SI-1 Conditions used to generate the simulated datasets. The cell parameters were kept fixed in all simulations.

Simulation number	Variation of occupancy	Active species
1	Linear	CO_2
2	Linear	CO_2 and Na
3	Linear	CO_2, Na21B (decreasing), Na21D (increasing)
4	Polynomial (from fit of refined data)	CO_2
5	Values obtained from refinement	CO_2, all Na^+ ions, Water

In PCA case 1 and 2, hkl intensities are given as input in the PCA (Figure S5 to S8). In these cases, by plotting the loadings of the reflections on PC1 vs. PC2 (top left figures) we obtain a scatter plot showing which reflections (indicated by sequential numbers) have the major weight on the two principal components. By plotting the loadings on two separate plots (top right figures) we have a representation of the variance according to the corresponding PC (y axis) of each reflection (x axis). Scores plot (bottom figures) represent the trends found in the data by PCA. In the bottom left figure we show the bi-plot of the scores of PC1 vs. scores of PC2. Each point represents one dataset (corresponding to one temperature) and the plot evidences the relationship between the dataset and both the PCs. The plot in the bottom right figure shows the score of each dataset (x axis) on one particular PC giving a representation of the trends (the variance) that is extracted from the data by that particular PC.

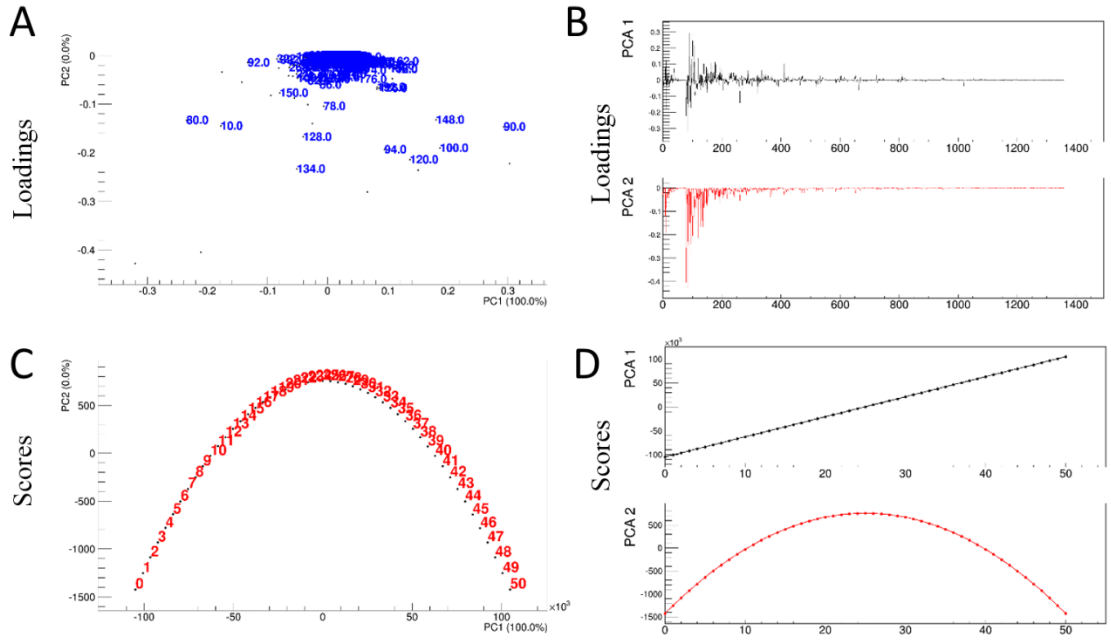

Figure S5 PCA results for simulated data from Simulation 1 (Linear variation of CO2). The values of the first two PCA eigenvalues are $\lambda1= 99.99\%$ $\lambda2=0.01\%$. A) Scatter plot of the PC2 vs PC1 hkl loadings, each point represents a reflection; B) Plot of the PC1 (black) and PC2 (red) loadings vs. reflection number; C) Scatterplot of the PC2 vs PC1 scores; D) plot of the PC1 (black) and PC1 (red) vs. dataset number.

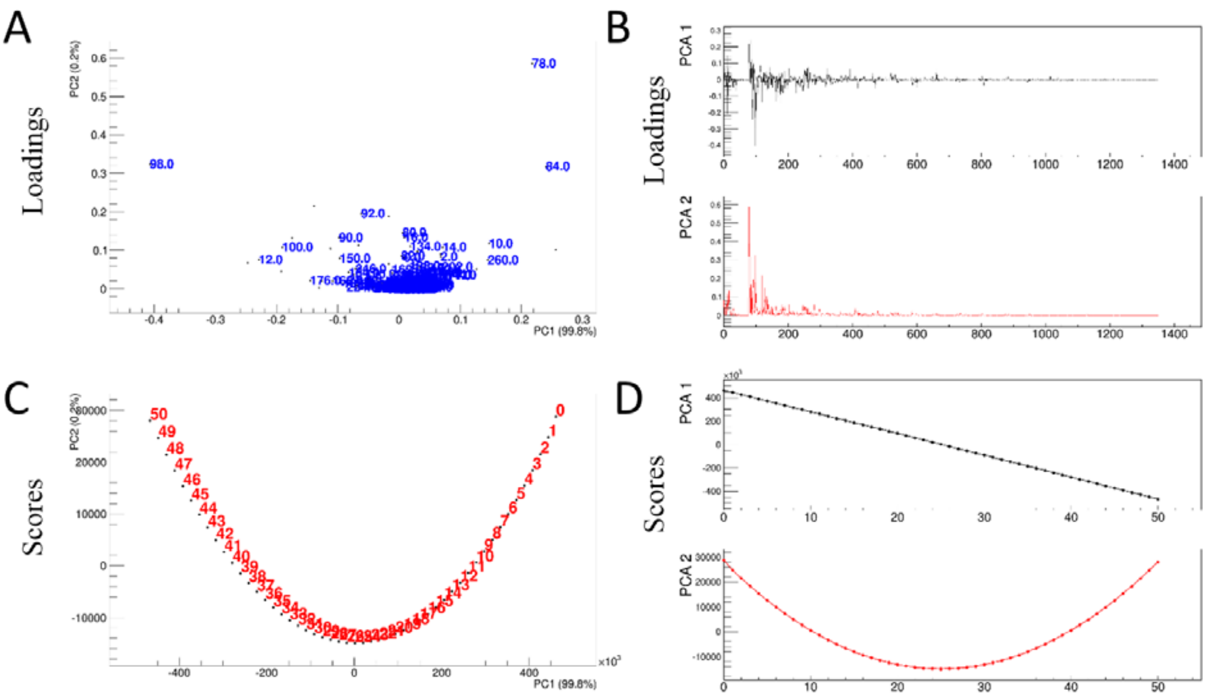

Figure S6 PCA results for simulated data from Simulation 2 (Linear and synchronized variation of CO2 and Na). The values of the first two PCA eigenvalues are λ1= 99.8% λ2=0.2%%. A) Scatter plot of the PC2 vs PC1 hkl loadings, each point represents a reflection; B) Plot of the PC1 (black) and PC2 (red) loadings vs. reflection number; C) Scatterplot of the PC2 vs PC1 scores; D) plot of the PC1 (black) and PC1 (red) vs. dataset number.

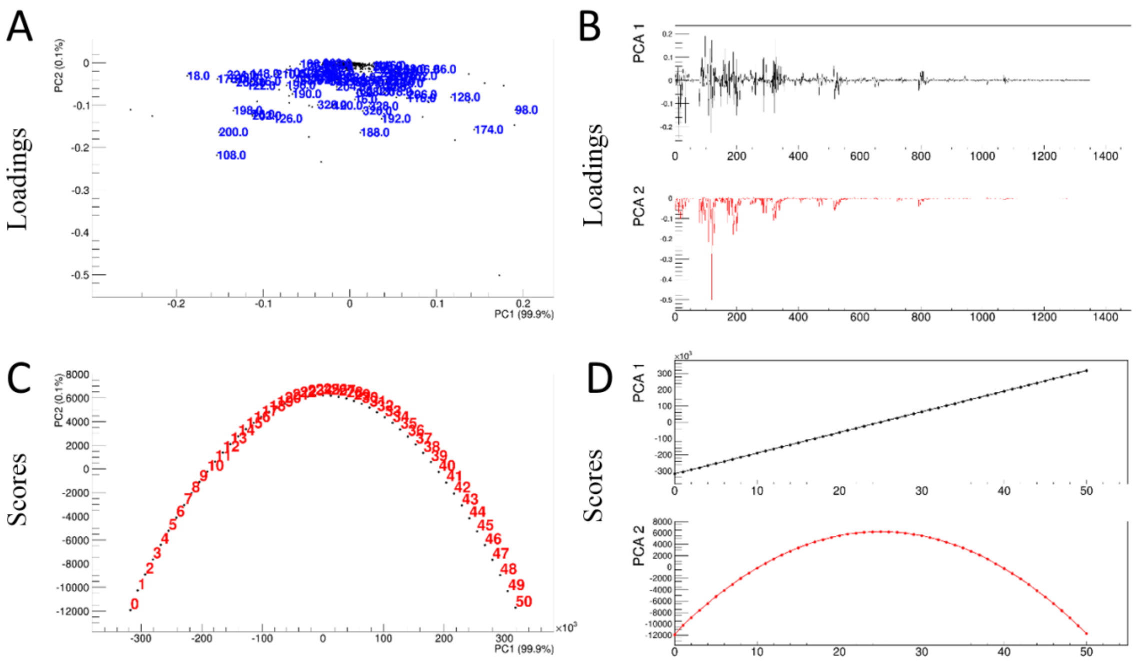

Figure S7 PCA results for simulated data from Simulation 3 (Linear variation of CO2, Na21B (decreasing), Na21D (increasing)) The values of the first two PCA eigenvalues are λ1= 99.8% λ2=0.2%%. A) Scatter plot of the PC2 vs PC1 hkl loadings, each point represents a reflection; B) Plot of the PC1 (black)

and PC2 (red) loadings vs. reflection number; C) Scatterplot of the PC2 vs PC1 scores; D) plot of the PC1 (black) and PC1 (red) vs. dataset number.

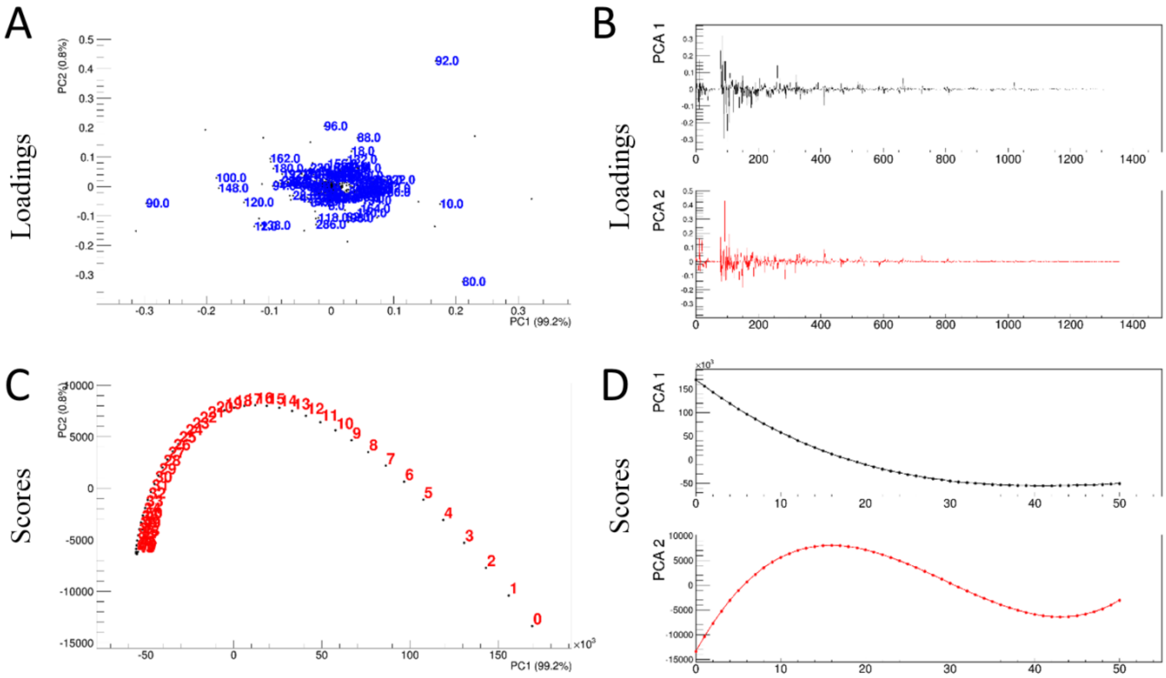

Figure S8 PCA results for simulated data from Simulation 4 (polinomial variation of CO_2). The values of the first two PCA eigenvalues are $\lambda1= 99.25\%$ $\lambda2=0.75\%$. A) Scatter plot of the PC2 vs PC1 hkl loadings, each point represents a reflection; B) Plot of the PC1 (black) and PC2 (red) loadings vs. reflection number; C) Scatterplot of the PC2 vs PC1 scores; D) plot of the PC1 (black) and PC1 (red) vs. dataset number.

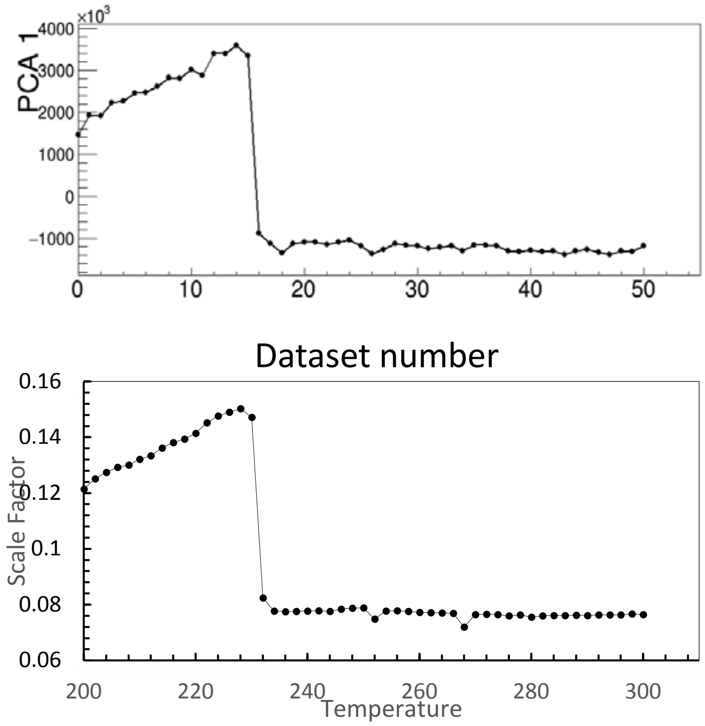

Figure S9 Scores of PC1 (top) obtained by PCA analysis of raw data compared with the plot of the scale factor obtained by structure refinement in TOPAS (bottom)

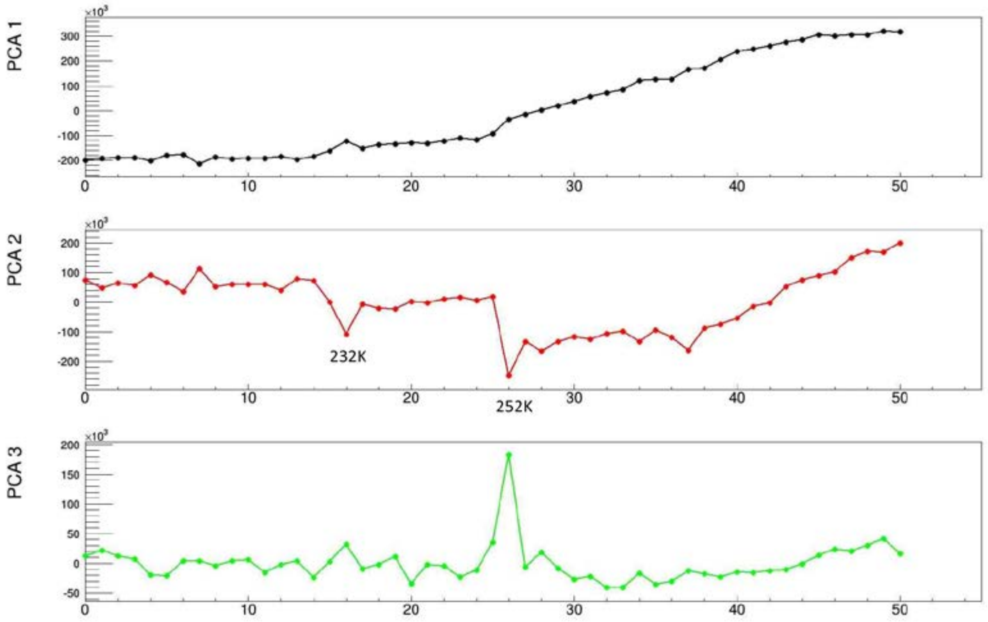

Figure S10 Scores plot from the first run of RootProf on the rescaled data showing the presence of two outliers ad 232K and 252K. The outlier ad 232K is due to the synchrotron refill as the refill occurred during the measure of this dataset. The outlier ad 252K after and inspection of the data reduction statistics

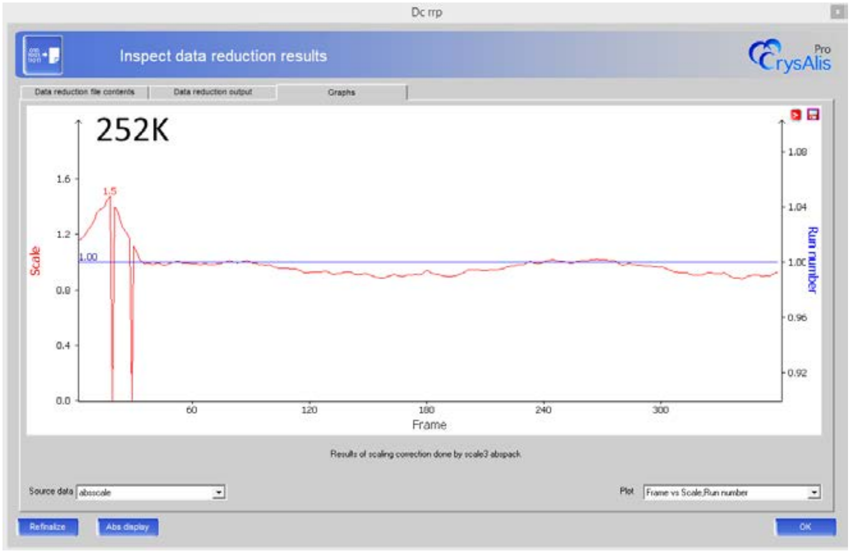

Figure S11 Screenshot of the absscale parameter vs. frame in the 252K dataset showing an anomalous profile.

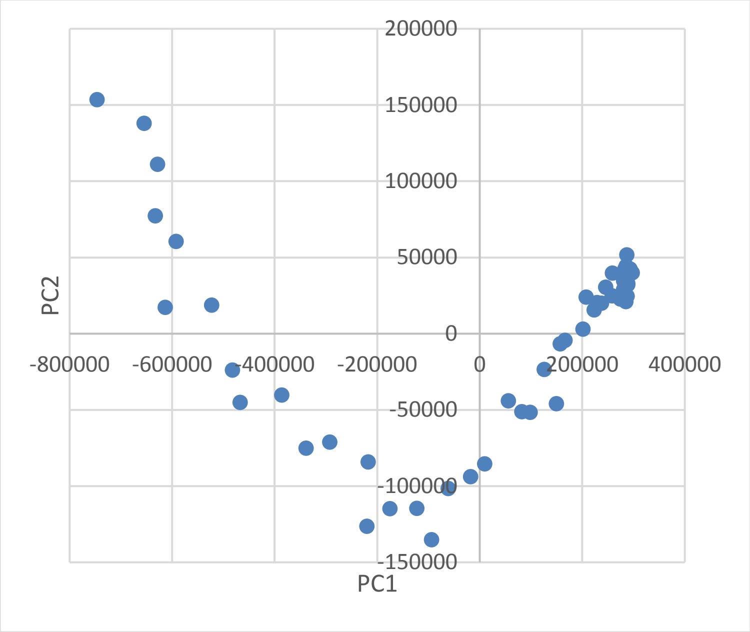

Figure S12 PCA analysis of Simulation 5. Scatter plot of PC2 vs PC1 scores.

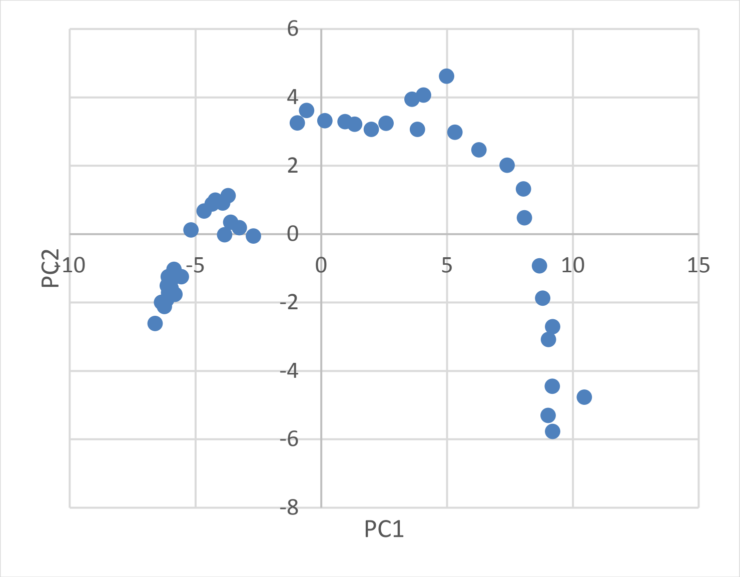

Figure S13 PCA analysis of real data. Scatterplot of PC2 vs PC1 scores

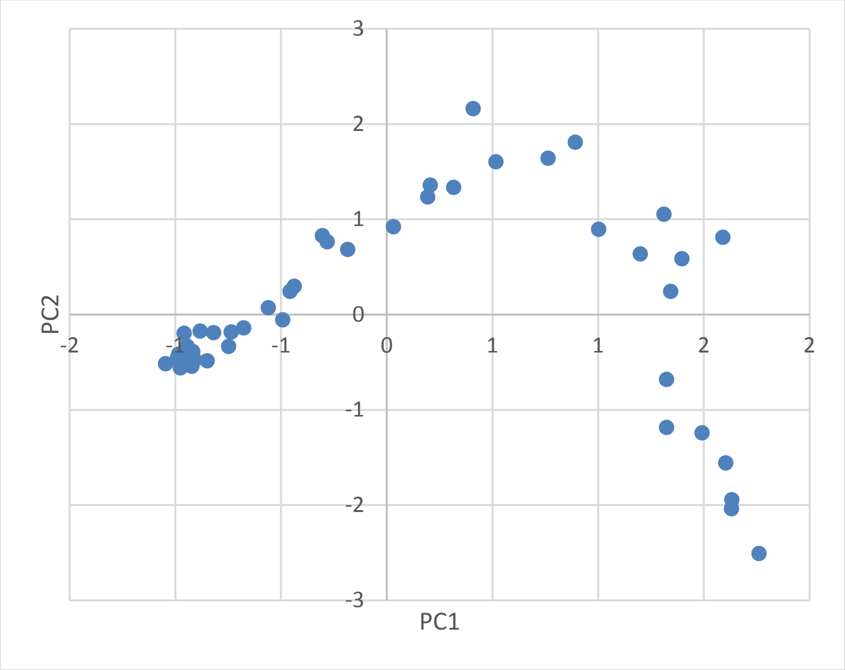

Figure S14 PCA analysis of occupancies. Scatter plot of PC2 vs PC1 scores.

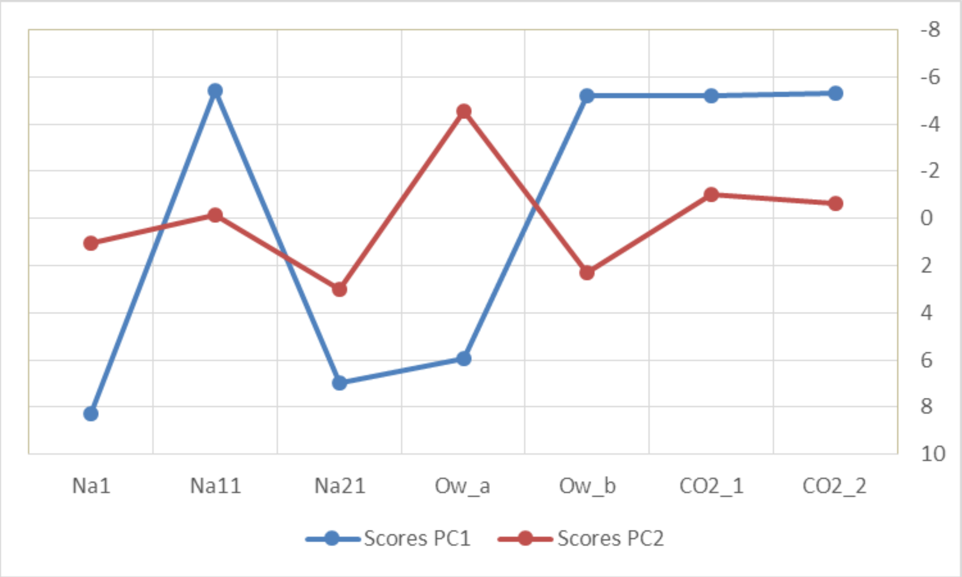

Figure S15 Plot of the scores from PCA applied to occupancy values (see also Figure 3c and Figure 4 in the manuscript) highlighting the correlation between the active species and the PCs.

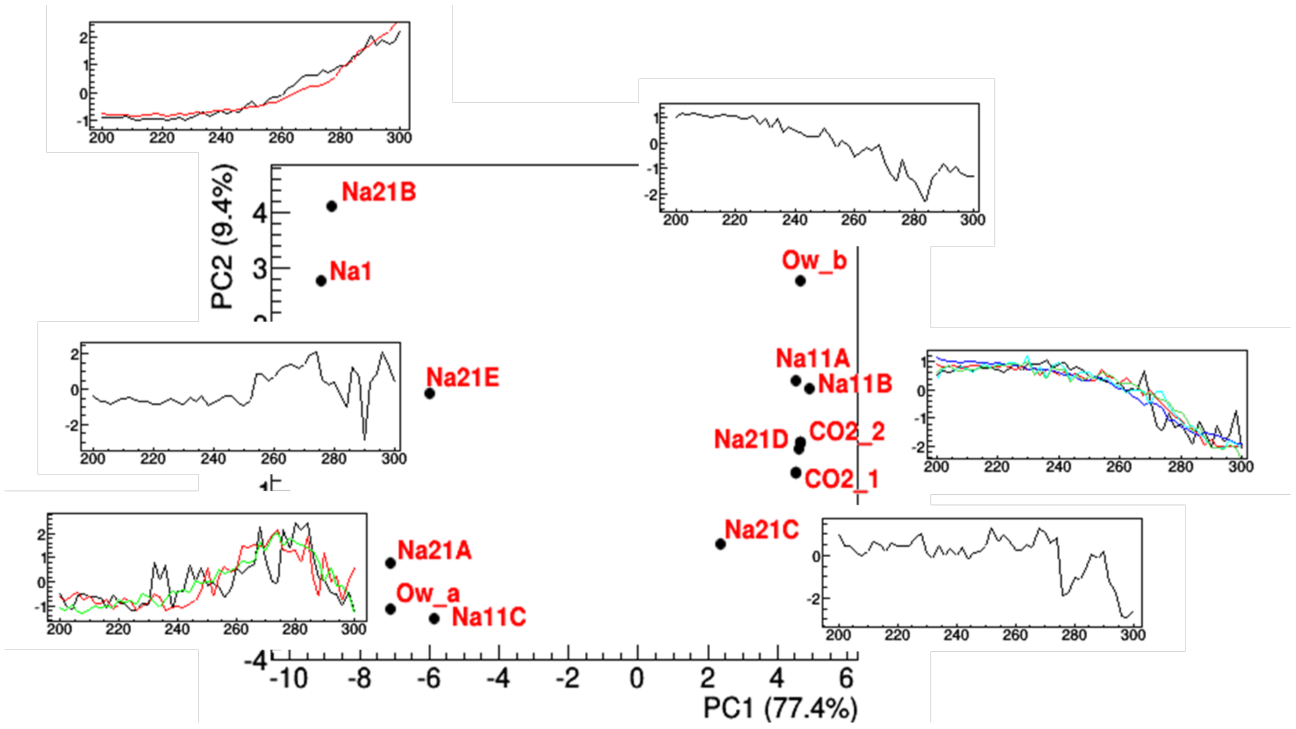

Figure S16 Scatter plot of PC2 vs. PC1 scores from PCA applied to occupancy values (separated by position) obtained from the sequential refinement. Insets show the normalized trends of occupancy, grouped according to the representative points in the score plot.

S5. Determination of the thermodynamic parameters.

The approach used by Garrone et al.(Garrone & Areán, 2005; Garrone *et al.*, 2017) for *in situ* IR data was extended to *in situ* single crystal X-ray diffraction data. Equilibrium constant was obtained by the Langmuir equation (Eq.1)

$$\frac{\theta}{1-\theta} = Kp \qquad Eq.1$$

The variable θ, i.e. the amount of adsorbed CO_2, was obtained by two methods. At first the sum of the CO_2 occupancies from structural refinement vs. 1/T was used since it is a direct measurement of the absorbed CO_2. Of course, this method requires the knowledge of the structure to carry out the refinement. Then the PC1 scores vs. 1/T trend was used, in the assumption that it describes uniquely the variance due to CO_2 adsorption. As discussed earlier, the method has the great advantage of being fast without any *a priori* knowledge of the system. As a drawback, PC scores are not determined in absolute scale and the have a sign

ambiguity, which limits their use in this case. The thermodynamic parameters were obtained by the van't Hoff equation (Eq.2):

$$\ln\left(\frac{\theta}{1-\theta}\right) = -\frac{\Delta H°}{R}\left(\frac{1}{T}\right) + \frac{\Delta S°}{R} \qquad Eq. 2$$

The slope and the intercept of the plot of left side of Eq. 2 vs 1/T allow the calculation of the enthalpy and entropy of the process. In particular, we used $\theta = \frac{occCO_2}{occCO_2^{max}}$ where $occCO_2$ are the values of the refined occupancy of CO_2 from the XRD experiment and $\theta = \frac{scorePC1}{scorePC1^{max}}$ where scorePC1 are the scores of the first principal component from the PCA. When PC scores are used, due to the arbitrariness of the sign, the right sign has to be assigned to the scores by a priori knowledge of the type of reaction.

		Occupancies															
Temp [K}	a=b=c	Na11_a	Na11_b	Na11_c	Na1	Na21_a	Na21_b	Na21_c	Na21_d	Na21_e	Ow_a	Ow_b	O1C C1C	O4C	C2CC	R1	Rall
300	24.86059	0.071	0.383	0.128	0.344	0.240	0.405	0.053	0.019	0.058	0.128	0.056	0.076	0.094	0.116	8.11	9.96
290	24.87647	0.060	0.403	0.139	0.340	0.239	0.350	0.061	0.056	0.052	0.187	0.074	0.093	0.098	0.122	8.06	9.82
280	24.90291	0.069	0.435	0.145	0.310	0.241	0.288	0.067	0.092	0.054	0.260	0.093	0.125	0.117	0.128	7.85	9.6
270	24.927	0.089	0.476	0.136	0.257	0.237	0.238	0.064	0.133	0.070	0.275	0.136	0.179	0.167	0.133	7.52	9.43
260	24.93817	0.096	0.506	0.134	0.222	0.235	0.212	0.063	0.152	0.080	0.258	0.170	0.200	0.209	0.133	7.38	9.32
250	24.94155	0.098	0.534	0.125	0.193	0.234	0.216	0.057	0.165	0.079	0.232	0.189	0.207	0.231	0.129	7.35	9.21
240	24.94853	0.101	0.551	0.123	0.170	0.232	0.206	0.057	0.174	0.084	0.223	0.197	0.216	0.258	0.127	7.44	9.28
230	24.94692	0.103	0.563	0.120	0.157	0.233	0.203	0.052	0.184	0.086	0.213	0.205	0.208	0.272	0.126	7.33	8.72
220	24.94745	0.099	0.578	0.117	0.149	0.234	0.207	0.049	0.183	0.084	0.203	0.210	0.207	0.279	0.124	7.23	8.51
210	24.94853	0.101	0.578	0.119	0.144	0.236	0.207	0.049	0.180	0.087	0.201	0.213	0.211	0.277	0.123	7.31	8.83
200	24.94919	0.095	0.585	0.114	0.148	0.238	0.207	0.049	0.180	0.084	0.194	0.213	0.210	0.272	0.125	7.29	8.74

Table S1 Data from manual refinement with SHELX.

Data #	Temp [K]	Na11A	Na11B	Na11C	Na1	Na21A	Na21B	Na21c	Na21D	Na21E	Ow_a	Ow_b	C1C	O4C
0	200	0.214	0.568	0.163	0.180	0.061	0.278	0.072	0.192	0.072	0.177	0.294	0.356	0.257
1	202	0.218	0.562	0.157	0.177	0.060	0.278	0.067	0.185	0.071	0.171	0.299	0.362	0.263
2	204	0.217	0.561	0.154	0.180	0.061	0.277	0.067	0.183	0.071	0.179	0.297	0.366	0.267
3	206	0.218	0.561	0.162	0.180	0.061	0.277	0.063	0.188	0.070	0.169	0.298	0.360	0.263
4	208	0.223	0.558	0.163	0.181	0.060	0.276	0.062	0.182	0.071	0.165	0.297	0.358	0.265
5	210	0.220	0.559	0.161	0.176	0.061	0.272	0.064	0.185	0.071	0.173	0.296	0.361	0.265
6	212	0.218	0.561	0.161	0.168	0.059	0.274	0.069	0.188	0.071	0.178	0.295	0.360	0.262
7	214	0.222	0.558	0.161	0.173	0.059	0.277	0.067	0.186	0.071	0.175	0.296	0.363	0.266
8	216	0.221	0.556	0.155	0.175	0.060	0.277	0.064	0.186	0.071	0.179	0.297	0.365	0.267
9	218	0.221	0.553	0.162	0.172	0.058	0.278	0.068	0.185	0.071	0.176	0.296	0.362	0.264
10	220	0.219	0.555	0.158	0.176	0.059	0.276	0.067	0.186	0.070	0.190	0.296	0.366	0.267
11	222	0.217	0.553	0.157	0.169	0.060	0.273	0.066	0.186	0.070	0.186	0.294	0.366	0.267
12	224	0.221	0.553	0.154	0.171	0.059	0.276	0.067	0.185	0.070	0.189	0.294	0.368	0.268
13	226	0.225	0.551	0.154	0.180	0.058	0.279	0.071	0.177	0.071	0.180	0.296	0.368	0.269
14	228	0.218	0.550	0.157	0.166	0.059	0.276	0.072	0.184	0.070	0.200	0.289	0.367	0.266
15	230	0.225	0.542	0.157	0.181	0.059	0.278	0.064	0.177	0.070	0.195	0.294	0.372	0.273
16	234	0.225	0.541	0.170	0.201	0.061	0.280	0.067	0.164	0.071	0.175	0.294	0.358	0.266
17	236	0.229	0.542	0.178	0.184	0.058	0.283	0.062	0.177	0.072	0.190	0.283	0.346	0.258
18	238	0.221	0.539	0.155	0.191	0.058	0.288	0.066	0.174	0.070	0.205	0.288	0.363	0.267
19	240	0.224	0.535	0.164	0.201	0.059	0.287	0.062	0.165	0.070	0.203	0.284	0.361	0.269
20	242	0.226	0.527	0.164	0.195	0.058	0.292	0.066	0.170	0.071	0.207	0.283	0.360	0.264
21	244	0.219	0.533	0.178	0.203	0.059	0.290	0.060	0.165	0.072	0.202	0.280	0.342	0.257
22	246	0.222	0.522	0.169	0.197	0.060	0.294	0.064	0.168	0.072	0.226	0.280	0.352	0.261
23	248	0.220	0.516	0.177	0.219	0.062	0.297	0.064	0.158	0.070	0.222	0.280	0.345	0.262
24	250	0.214	0.515	0.167	0.239	0.067	0.303	0.067	0.154	0.070	0.222	0.286	0.350	0.262
25	254	0.211	0.523	0.166	0.223	0.065	0.306	0.069	0.177	0.078	0.247	0.271	0.356	0.256
26	256	0.209	0.515	0.161	0.251	0.067	0.314	0.071	0.160	0.078	0.239	0.276	0.355	0.258
27	258	0.207	0.504	0.166	0.253	0.066	0.315	0.069	0.160	0.076	0.242	0.271	0.349	0.253

28	260	0.208	0.496	0.177	0.257	0.066	0.320	0.065	0.146	0.077	0.249	0.263	0.341	0.252
29	262	0.201	0.495	0.173	0.286	0.071	0.331	0.064	0.158	0.079	0.260	0.266	0.341	0.248
30	264	0.203	0.483	0.177	0.298	0.071	0.338	0.067	0.147	0.079	0.259	0.271	0.341	0.250
31	266	0.202	0.475	0.178	0.321	0.071	0.350	0.066	0.140	0.080	0.260	0.269	0.338	0.249
32	268	0.219	0.460	0.198	0.334	0.071	0.357	0.075	0.114	0.079	0.295	0.273	0.325	0.248
33	270	0.196	0.466	0.181	0.336	0.071	0.364	0.073	0.129	0.080	0.278	0.260	0.327	0.239
34	272	0.170	0.463	0.169	0.337	0.073	0.364	0.068	0.111	0.082	0.306	0.250	0.321	0.248
35	274	0.166	0.453	0.168	0.357	0.074	0.370	0.070	0.099	0.083	0.314	0.243	0.318	0.248
36	276	0.181	0.426	0.196	0.344	0.070	0.378	0.044	0.081	0.076	0.289	0.261	0.314	0.238
37	278	0.170	0.424	0.187	0.362	0.070	0.396	0.044	0.068	0.075	0.302	0.247	0.301	0.233
38	280	0.172	0.410	0.200	0.371	0.070	0.418	0.052	0.059	0.075	0.295	0.243	0.300	0.228
39	282	0.163	0.406	0.197	0.371	0.068	0.432	0.052	0.042	0.072	0.291	0.235	0.291	0.224
40	284	0.153	0.395	0.200	0.407	0.073	0.446	0.056	0.039	0.069	0.286	0.226	0.274	0.224
41	286	0.174	0.390	0.182	0.415	0.064	0.473	0.063	0.025	0.079	0.277	0.247	0.278	0.220
42	288	0.165	0.396	0.171	0.431	0.061	0.480	0.061	0.002	0.077	0.260	0.251	0.265	0.216
43	290	0.151	0.395	0.170	0.483	0.070	0.496	0.064	0.014	0.061	0.224	0.258	0.249	0.210
44	292	0.170	0.390	0.163	0.450	0.064	0.509	0.050	0.000	0.075	0.247	0.251	0.250	0.210
45	294	0.156	0.385	0.162	0.469	0.065	0.519	0.045	0.000	0.078	0.216	0.256	0.248	0.210
46	296	0.162	0.380	0.156	0.453	0.060	0.534	0.034	0.000	0.083	0.216	0.250	0.252	0.214
47	298	0.183	0.372	0.163	0.459	0.064	0.556	0.033	0.000	0.079	0.198	0.248	0.249	0.213
48	300	0.150	0.373	0.153	0.501	0.067	0.568	0.037	0.000	0.075	0.166	0.247	0.231	0.210

Table S2 Table SI-2 Data from TOPAS sequential refinement.
